# Effect of marker position and size on the registration accuracy of HoloLens in a non-clinical setting with implications for high-precision surgical tasks

**DOI:** 10.1007/s11548-021-02354-9

**Published:** 2021-04-15

**Authors:** Laura Pérez-Pachón, Parivrudh Sharma, Helena Brech, Jenny Gregory, Terry Lowe, Matthieu Poyade, Flora Gröning

**Affiliations:** 1 School of Medicine, Medical Sciences and Nutrition, University of Aberdeen, Aberdeen, UK; 2 School of Simulation and Visualisation, Glasgow School of Art, Glasgow, UK; 3 Head and Neck Oncology Unit, Aberdeen Royal Infirmary (NHS Grampian), Aberdeen, UK

**Keywords:** Image marker, Augmented reality, Image-guided surgery, Holographic headsets’ registration error

## Abstract

**Purpose:**

Emerging holographic headsets can be used to register patient-specific virtual models obtained from medical scans with the patient’s body. Maximising accuracy of the virtual models’ inclination angle and position (ideally, ≤ 2° and ≤ 2 mm, respectively, as in currently approved navigation systems) is vital for this application to be useful. This study investigated the accuracy with which a holographic headset registers virtual models with real-world features based on the position and size of image markers.

**Methods:**

HoloLens^®^ and the image-pattern-recognition tool Vuforia Engine™ were used to overlay a 5-cm-radius virtual hexagon on a monitor’s surface in a predefined position. The headset’s camera detection of an image marker (displayed on the monitor) triggered the rendering of the virtual hexagon on the headset’s lenses. 4 × 4, 8 × 8 and 12 × 12 cm image markers displayed at nine different positions were used. In total, the position and dimensions of 114 virtual hexagons were measured on photographs captured by the headset’s camera.

**Results:**

Some image marker positions and the smallest image marker (4 × 4 cm) led to larger errors in the perceived dimensions of the virtual models than other image marker positions and larger markers (8 × 8 and 12 × 12 cm). ≤ 2° and ≤ 2 mm errors were found in 70.7% and 76% of cases, respectively.

**Conclusion:**

Errors obtained in a non-negligible percentage of cases are not acceptable for certain surgical tasks (e.g. the identification of correct trajectories of surgical instruments). Achieving sufficient accuracy with image marker sizes that meet surgical needs and regardless of image marker position remains a challenge.

**Supplementary Information:**

The online version contains supplementary material available at 10.1007/s11548-021-02354-9.

## Introduction

**Fig. 1:**
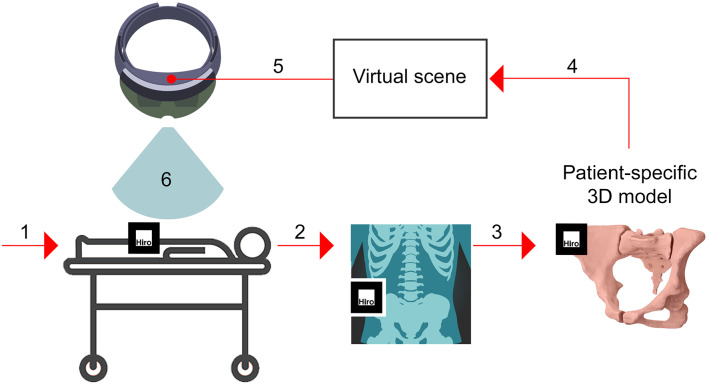
Workflow example of image overlay surgery with a holographic headset: (1) attachment of a radio-opaque image marker to the patient’s body surface; (2) scanning of the patient to obtain a 3D image dataset; (3) creation of a patient-specific virtual model from the images; (4) creation of a virtual scene including the virtual model and image marker position; (5) installation of an app on the headset that includes the virtual scene and image-pattern-recognition algorithms; and (6) overlay of the virtual model on the patient’s body surface

Emerging augmented reality (AR) technologies such as holographic headsets allow the overlay of patient-specific virtual models obtained from medical scans on the patient’s body surface in a predefined position [1]. This helps to transfer image data produced during the planning of the surgery (e.g. the correct trajectories of surgical instruments) to the operating room. Registration of virtual models to the patient’s body may be achieved by fixing fiducial markers (e.g. radio-opaque image markers) to the patient’s body at the time of scanning. During surgery, these image markers are recognised by image-pattern-recognition tools that compute the registration (Fig. 1). However, image marker detection and rendering stability, and thus registration accuracy, may be affected by several factors [2], e.g. image marker position and size. To use holographic headsets for surgical guidance, their accuracy must be equal or below that one of currently approved navigation systems, e.g. Brainlab™ or Medtronic StealthStation™ provide trajectory angle and positional errors of ≤ 2° and ≤ 2 mm, respectively [3, 4].

### Position and size of image markers

The position and size of image markers may affect the image marker pose estimated by computer vision systems [5] and thus the accuracy of the registration of virtual models with real-world features in AR applications. Uematsu et al. [6] argued that detection of image markers becomes unstable when they lie on the central axis of the camera view frustum. They attributed this effect to slight differences in the detection of image features (e.g. edges or corners) which may reduce the positional accuracy with which virtual models are rendered. In addition, achieving an optimal balance between image marker size, camera-to-image-marker distance and camera resolution is key to maximise registration accuracy. For example, previous research has demonstrated that the accuracy of image marker pose estimation is affected by the image marker size [7] and camera-to-image-marker distance and angle [8]. High-resolution cameras allow the use of small image markers and/or their location far from the camera while preserving their optimal detection and correct registration of the virtual model. For instance, the recommendation for the Vuforia Engine™ is a minimum width of image markers calculated by dividing the camera-to-image-marker distance by 10 [9], which must be adjusted based on the resolution of the camera used.

### Use of image markers in augmented-reality-based surgical guidance

The AR-based guidance of procedures such as bone sectioning [10], bone drilling [11] or the identification of correct entry points and trajectories of surgical instruments [12] requires high accuracy. Certain types of surgery, e.g. the excision of small tumours [13] or otologic surgery [14], require submillimetre accuracy. Some studies using image-pattern-recognition have achieved submillimetre accuracy [15–19]. Few studies have systematically analysed the accuracy that can be achieved with the combined use of image-pattern-recognition algorithms and holographic headsets [20]. These studies did not measure the effect of image marker position and size on registration accuracy [1]. However, understanding this effect is the key to implement these systems in clinical practice as low accuracy may lead to errors in the position and dimensions with which the virtual models are perceived by surgeons during surgery.

### Aim and objectives

This study explored the accuracy with which a holographic headset registers virtual models with real-world features using an image-pattern-recognition tool and discussed its implications for surgical guidance. The research questions were: (a) “What is the error in the position and dimensions of the rendered virtual models?” and (b) “What is the effect of image marker position and size on this error?” Our results are expected to help software developers and manufacturers to minimise errors and thus to validate the use of holographic headsets in clinical practice.

## Materials and methods

### Experimental setup

An AR app (App 1) for the holographic headset HoloLens^®^ (first generation) was created using Vuforia Engine™ (version 6.2.10) [21] and Mixed Reality Toolkit (version 1.5.8.0). The camera resolution of this headset is 2.4 megapixels [22]. App 1 allowed for the detection of a digital image marker (referred to as “marker” henceforth) by the headset’s camera. According to Vuforia Engine™, the marker’s score was 4 in a 1–5 scale that rates the quality of markers for their optimal detection [9]. Marker detection triggered the rendering of a virtual model (a 5-cm-radius virtual hexagon) on the headset’s lenses in a set position.

**Fig. 2:**
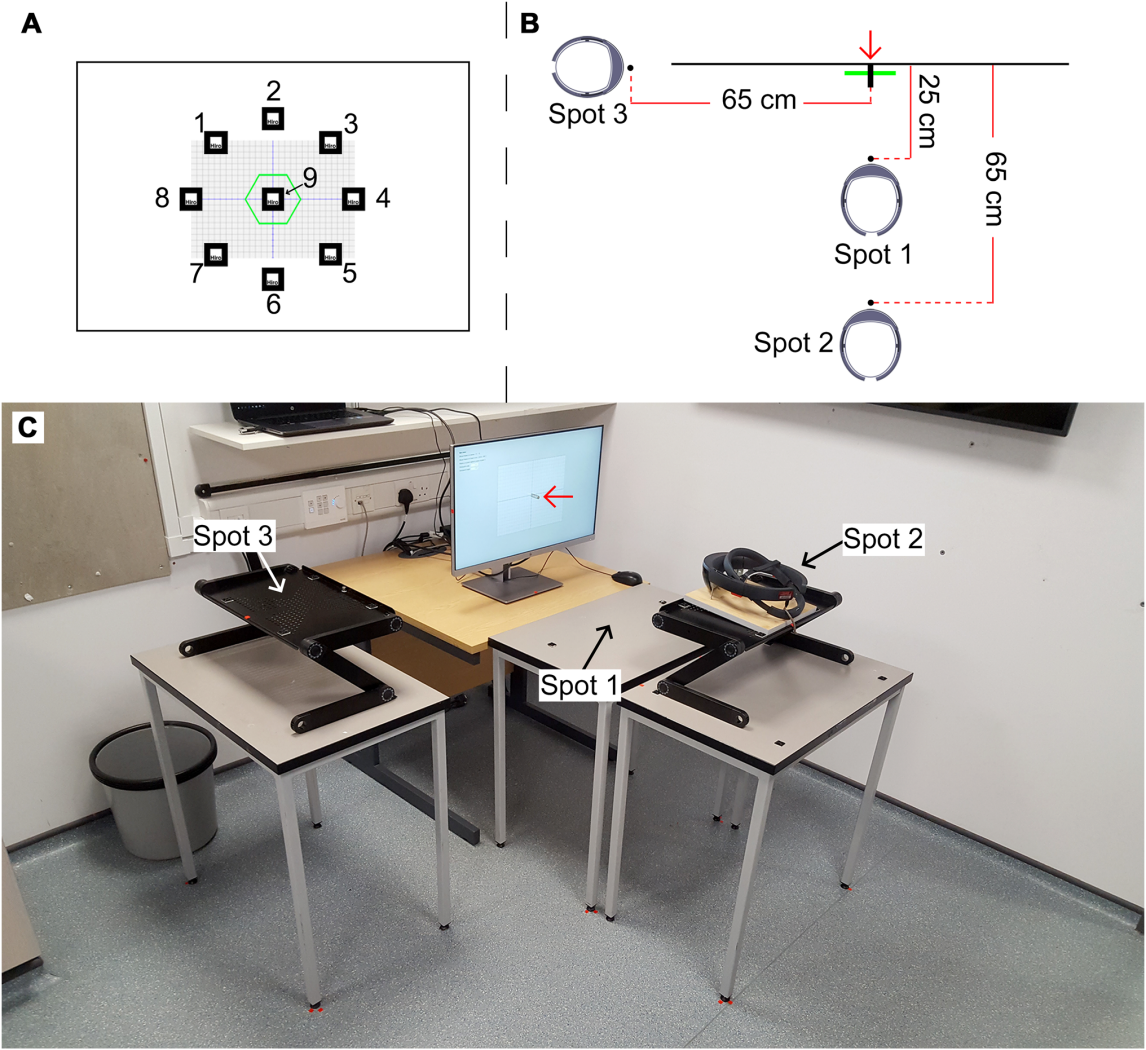
Frontal view of the experimental setup (**a**) showing a digital graph chart and digital image markers at positions 1–9 displayed on a monitor and a virtual hexagon rendered on the lenses of a holographic headset and captured on photographs taken with the headset’s camera; top view (**b**) depicting the virtual hexagon, a 10-mm-diameter cylinder (red arrow) attached to the monitor’s surface and aligned with the centre of the digital graph chart and headset at Spots 1–3; and perspective view (**c**) showing Spots 1–3 and the cylinder (red arrow). Digital image markers at position 9 were aligned with the centre of the digital graph chart and those at positions 1–8 were at 108 mm from the centre

A second app (App 2) was created for the display of the marker on a monitor at nine positions (Fig. 2) and in three sizes (4 × 4, 8 × 8 and 12 × 12 cm markers). This resulted in 27 markers displayed one at a time. These marker sizes allowed exploring the extent to which marker size increase affects the registration error. App 2 was executed on a laptop connected to the monitor. The headset’s camera was aligned with the centre of a digital graph chart displayed on the monitor to scale (280 × 200 mm). Three positions of the headset’s camera were set (Spots 1–3). Spot 1 was at 25 cm from the monitor’s surface and Spots 2 and 3 at 65 cm (Fig. 2). Marker detection by the headset’s camera was performed with the headset on Spot 1 (for the 4 × 4 cm markers) and 2 (for the 8 × 8 and 12 × 12 cm markers). Spot 1 was necessary as detection of 4 × 4 cm markers from distances further than 25 cm failed or the graphical rendering of the virtual hexagon was unstable.

To ensure the correct position of the headset, the headset was fixed to a board and the board was placed on one of two adjustable lecterns (depending on the experimental step) using plastic markers as positional references (Online Resource 1). The headset’s camera was positioned so that the centre of the photographs matched the centre of the digital graph chart. Adjustment was assessed using calibration photographs captured with the headset’s camera and measuring tape that was attached to the monitor’s surface and to its side as a reference (Online Resource 1). As the images captured by the headset’s camera may suffer distortion [23], a calibration photograph was taken from Spot 3 which included a framing square aligned with the centre of the monitor’s surface (Online Resource 2). The framing square was used to calculate a correction factor that was applied to the measurements extracted from photographs taken from Spot 3.

The use of a simple experimental setup allowed us to obtain a sufficiently large number of measurements and sample size for statistical testing. In addition, we chose to overlay the virtual models on the flat surface of a monitor rather than on a volumetric surface to minimise potential sources of bias (e.g. measurement errors due to a wide variety of angles between the virtual models and the real-world surface).

### Procedure

**Fig. 3:**
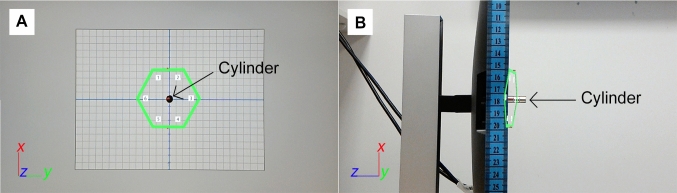
Photograph taken from Spot 2 (**a**) showing a virtual hexagon and digital graph chart and photograph taken from Spot 3 (**b**) showing the virtual hexagon and a cylinder stuck to the centre of the monitor’s surface and used as a reference for the measurement of the virtual hexagon’s y-axis inclination angle and its distance to the monitor’s surface

During the experiment, App 1 was launched for each marker detection to avoid cumulative errors in the mapping of the markers [24]. Once the headset’s camera detected a marker on the monitor, a virtual hexagon was rendered on the headset’s lenses. The marker was then removed from the monitor, and the virtual hexagon was captured on two photographs taken from Spots 2 and 3 under controlled lighting conditions (i.e. in a room without windows to avoid shifts caused by natural light). The headset was moved from Spots 1 to 2 to capture the first photograph (for the 4 × 4 cm marker) and from Spots 2 to 3 to capture the second photograph (for all markers). Photographs showed orthogonal (Spot 2) and lateral (Spot 3) views of the virtual hexagon (Fig. 3). Assuming a correct system’s performance, the virtual hexagon appeared on the photographs as overlaid on the monitor’s surface and aligned with the centre of the digital graph chart. Six repetitions of this process were done following the results of a power analysis for an ANOVA using G*Power [25]. Analysis of errors associated with 4 × 4 cm markers was possible for markers at position 9 only (Fig. 2), as markers at positions 1–8 lay outside the field of view of the headset’s camera due to the headset’s proximity to the monitor’s surface (25 cm) and thus their detection was not possible.

### Data extraction and analysis

The virtual hexagons’ vertices for all experimental conditions (*n* = 5832) were measured on the photographs taken from Spot 2 using the digital graph chart as a reference. The virtual hexagons’ y-axis inclination angle (henceforth “inclination angle”) and distance to the monitor’s surface (henceforth “distance-to-monitor”) were measured on the photographs taken from Spot 3 (*n* = 972) using the cylinder as a reference (Fig. 3). The measurements were taken by three researchers (LP, PS and HB) aged 20–35 years who repeated data collection three times. The intra-class correlation coefficient (ICC) was used to analyse the intra- and inter-observer variability [26]. The positions of the virtual hexagons’ vertices were used to calculate the virtual hexagons’ centroid position and area. Errors (i.e. the difference between measured and predicted values) were calculated for the following variables: inclination angle, distance-to-monitor, vertex and centroid positions and area.

SPSS 25 (IBM Statistics, Chicago, USA) and Sigma Plot 14 (Systat Software, San Jose, CA) were used for statistical analysis. Errors exceeding 1.5 and 3 times the interquartile range were classified as weak and strong outliers, respectively [27]. Errors were compared across groups within each independent variable (i.e. marker positions 1–9 and 4 × 4, 8 × 8 and 12 × 12 cm markers). Since data were not normally distributed, an ANOVA could not be performed and thus the nonparametric Mann–Whitney *U* and Kruskal–Wallis *H* tests (*p* < 0.001) were used for comparisons between two groups and more than two groups, respectively. Turkey post hoc tests (*p* < 0.05) were used for pairwise comparisons, except to compare the vertex position between different marker positions, for which Dunn’s method (*p* < 0.05) was used. Correlations were obtained using Pearson’s correlation coefficient. To correlate the vertex position error (*n* = 5832) with the inclination angle and distance-to-monitor errors, sample size for all these variables was matched by calculating the average vertex position of each virtual hexagon (*n* = 972). Several error categories for the inclination angle (≤ 1, ≤ 2 and ≤ 5°) and vertex and centroid position (≤ 1, ≤ 2 and ≤ 5 mm) were determined. The percentage of cases within each category was calculated.

## Results

The results show a small intra-observer variability (ICC = 0.9), i.e. high similarity between measurements repeated by the same researcher (Online Resource 3). The inter-observer variability in the measurement of the inclination angle, distance-to-monitor and vertex position was low: < 0.5°, < 1 mm and < 0.1 mm, respectively (Online Resources 4, 5 and 6). Errors for all dependant variables analysed in this study are presented in Table 1. Strong outliers are presented in Online Resource 7.

**Table 1:** Mean errors for all dependant variables including all marker positions (1–9) and 8 × 8 and 12 × 12 cm markers

		*N*	Min	Max	Mean	SD
Inclination angle (°)		972	0	13.6	4.2	2.8
Distance-to-monitor (mm)		972	0	32.9	10.8	7.5
Vertex position (mm)		5832	0	14.8	2.1	2.1
Centroid position (mm)		972	0	9.0	1.7	1.7
Area (%)	Absolute	972	0	15.5	3.0	3.1
	Relative	972	− 15.5	5.8	− 0.8	4.3

Inclination angle and distance-to-monitor errors were expected to affect the virtual hexagons’ position and dimensions when observed from an orthogonal view (Online Resource 8). This was indeed the case for the distance-to-monitor error (Online Resource 9). However, correlations between the inclination angle error and virtual hexagons’ position and dimensions were significant but weak (*r* ≤ 0.4). In addition, the system rendered the virtual hexagons more often in front of the monitor’s surface, and thus closer to the headset’s camera, than behind it (Online Resource 10).

### Effect of marker position

**Fig. 4:**
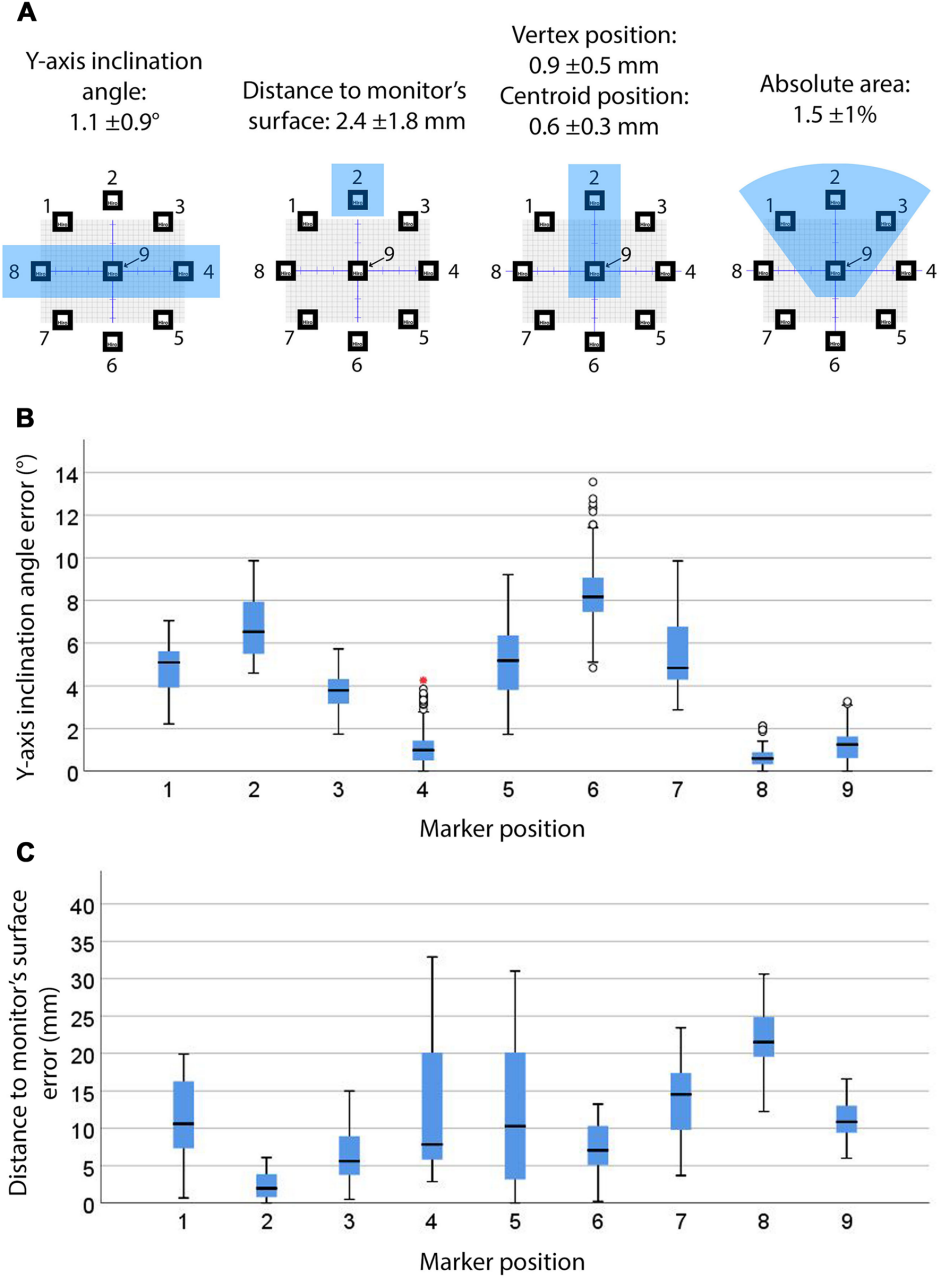
Marker positions with significantly smallest errors (*p* < 0.05) for all dependant variables (highlighted with blue shading) and their mean errors (**a**), inclination angle (**b**, *n* = 972) and distance-to-monitor (**c**, *n* = 972) errors for 8 × 8 and 12 × 12 cm markers at marker positions 1–9. Whiskers represent the maximum and minimum values. Weak outliers are indicated with circles and strong outliers with red asterisks

**Fig. 5:**
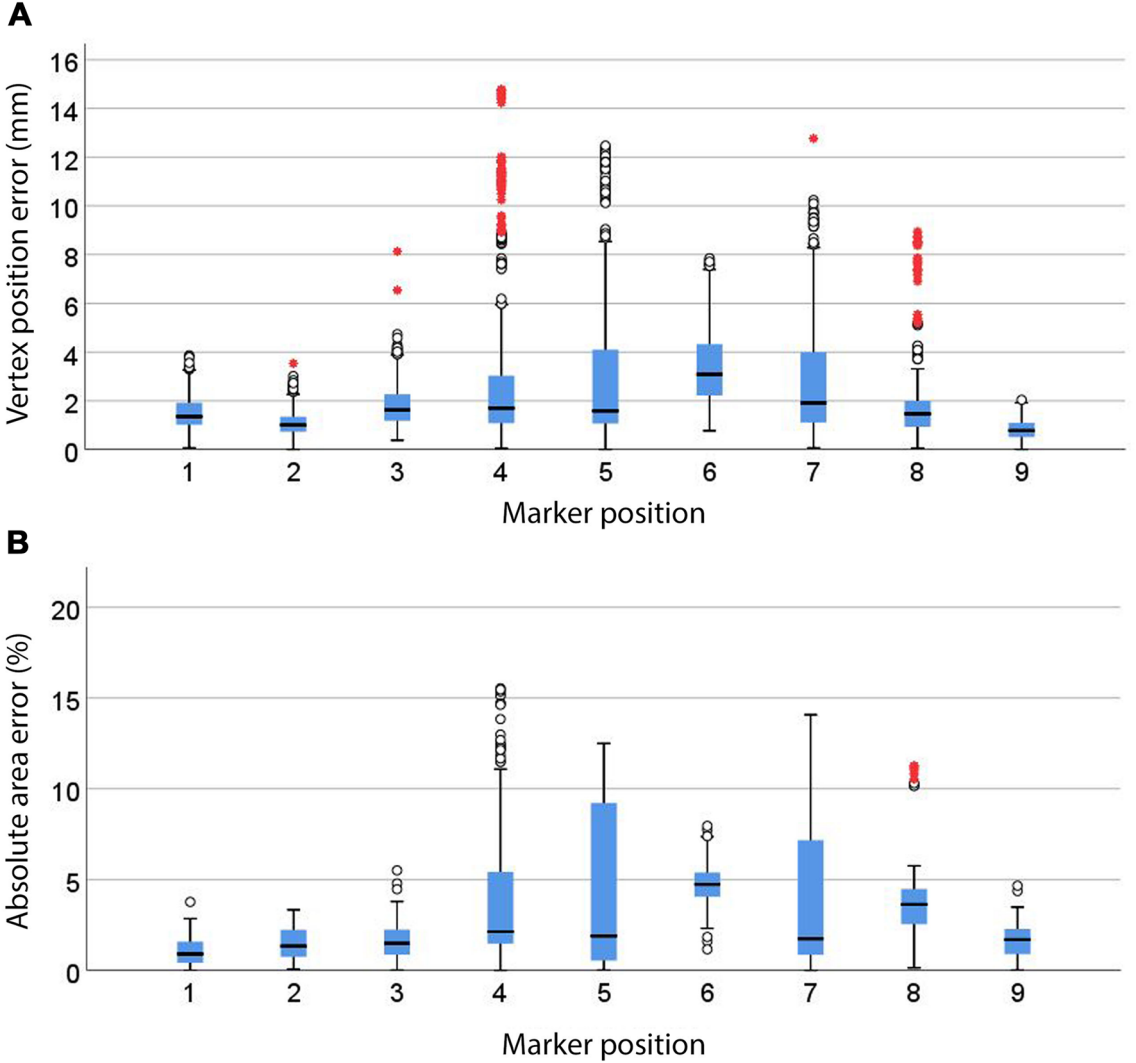
Vertex position (**a**, *n* = 5832) and absolute area (**b**, *n* = 972) errors for 8 × 8 and 12 × 12 cm markers and marker positions 1–9 (*p* < 0.05). Whiskers represent the maximum and minimum values. Weak outliers are indicated with circles and strong outliers with red asterisks

Figure 4a shows the marker positions associated with the smallest errors in the virtual hexagons’ position and dimensions. The inclination angle, distance-to-monitor and area errors significantly differed across all marker positions (*p* < 0.001). Marker positions at the level of the headset’s camera (i.e. 4, 8 and 9) presented significantly smaller inclination angle errors (*p* < 0.05), as shown in Fig. 4b. These marker positions, along with marker position 6 (i.e. below the level of the headset’s camera and in line with its y-axis), presented numerous outliers thus suggesting inconsistent registration of the inclination angle. Marker position 2 (i.e. above the level of the headset’s camera and in line with its y-axis) provided the smallest distance-to-monitor error (*p* < 0.05) as shown in Fig. 4c. Marker position 9 (which laid on the central axis of the camera view frustum) provided the smallest vertex and centroid position errors (Fig. 5a and Online Resource 11), followed by marker position 2 (*p* < 0.05). Numerous outliers indicate inconsistent vertex registration across all marker positions. Marker positions 1, 2, 3 and 9 (i.e. laying on the central axis of the camera view frustum or above) showed significantly smaller absolute area errors (*p* < 0.05), as shown in Fig. 5b. Relative area errors with marker position 6 showed a significant reduction of the area (*p* < 0.05) compared to the other marker positions (Online Resource 11).

### Effect of marker size

**Fig. 6:**
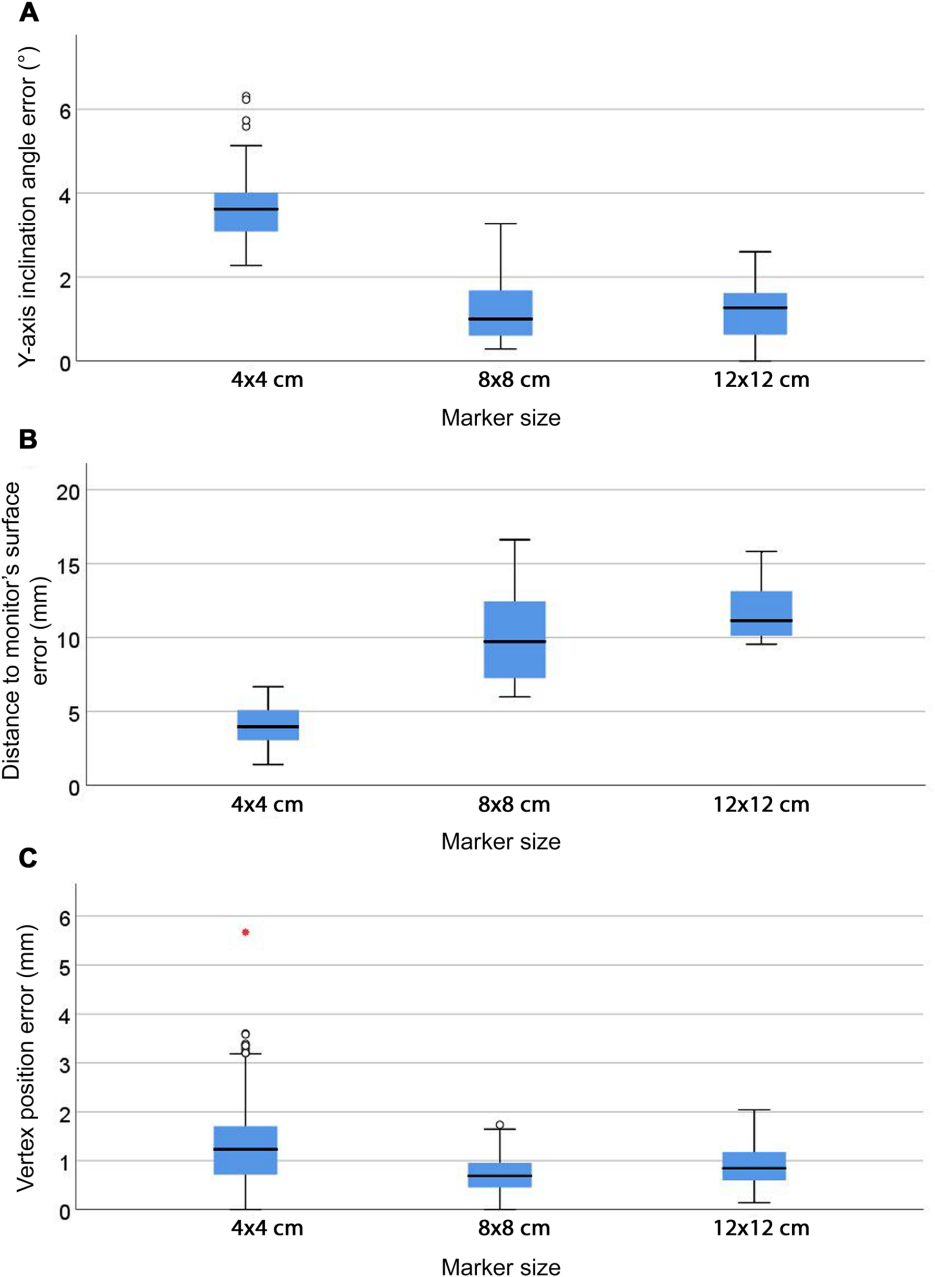
Inclination angle (**a**), distance-to-monitor (**b**) and vertex position (**c**) errors (*p* < 0.05) for 4 × 4, 8 × 8 and 12 × 12 cm markers at marker position 9 (*n* = 162). Whiskers represent the maximum and minimum values. Weak outliers are indicated with circles and strong outliers with red asterisks

Compared to 8 × 8 cm markers, 12 × 12 cm markers provided significantly smaller errors for all variables (*p* < 0.001), as shown in Online Resource 12. Comparison of the three marker sizes (i.e. 4 × 4, 8 × 8 and 12 × 12 cm) was possible for marker position 9 only, as this was the sole marker position providing data for 4 × 4 cm markers. Larger inclination angle and smaller distance-to-monitor errors (*p* < 0.05) were found for 4 × 4 cm markers (Fig. 6). The smallest vertex and centroid position and area errors (*p* < 0.05) were found for 8 × 8 cm markers (Fig. 6c and Online Resource 13). Centroid position errors remained ≤ 0.5 mm with 8 × 8 and 12 × 12 cm markers, whereas they increased to 1.21 mm with 4 × 4 cm markers. The absolute area error for 4 × 4 cm markers was smaller than for 8 × 8 and 12 × 12 cm markers, but relative values showed a reduction in the area for 4 × 4 cm markers (Online Resource 13).

### Percentage of errors

Most inclination angle errors (61.6%) were ≤ 5°, with 38.4% being 5–10° (Table 2). 70.7% and 76% of vertex and centroid position errors were ≤ 2° and 2 mm, respectively, with a vast majority of them being ≤ 5 mm.

**Table 2:** Percentage of errors in the virtual hexagons’ position and dimensions for 8 × 8 and 12 × 12 cm markers at marker positions 1–9 (*n* = 972)

	Error	%
Inclination angle (°)	≤ 1	20.6
	≤ 2	30.2
	≤ 5	61.6
Vertex position (mm)	≤ 1	20.9
	≤ 2	70.7
	≤ 5	91.3
Centroid position (mm)	≤ 1	45.1
	≤ 2	76
	≤ 5	94

## Discussion

To the authors’ knowledge, this is the first study measuring the effect of marker position and size on the accuracy with which a holographic headset registers virtual models with real-world features. Our results highlight the risk of large errors in the position and dimensions of virtual models as observed by users wearing the headset. These results are expected to help manufacturers and software developers to minimise registration errors and thus validate the use of holographic headsets in clinical practice.

### Error in the position and dimensions of the virtual models

Mean vertex and centroid position errors were up to 5 mm (Table 1). These findings are in line with the results of a systematic review which explored AR-guided open surgery [20] and with previous studies using holographic headsets [28–32]. A non-negligible percentage of cases (24–30% approximately) presented positional errors over 2 mm (Table 2). Similarly, mean errors of 4.2 ± 2.8° in the inclination angle of virtual models (Table 1) are in line with previous research exploring AR-guided bone tissue sectioning. For instance, Pietruski et al. [10] reported errors of 4.2 ± 1° and 5.4 ± 3.9° in sagittal and frontal osteotomy planes and Viehöfer et al. [33] found similar errors of 4.9 ± 4.2°. Smaller errors of 2 ± 1.2° and 1.3 ± 1.2° were reported in recent studies [34, 35]. In our study, only 30.2% of cases presented ≤ 2° inclination angle errors (Table 2).

### Effect of image marker position and size

Small inclination angle, distance-to-monitor and vertex and centroid position errors were restricted to specific marker positions and sizes (Figs. 4, 5 and 6). This shows that general guidelines provided by developers of image-pattern-recognition tools may not suit the purpose of a particular AR app. According to the Vuforia Engine™ guidelines [9], the minimum marker size for an optimal marker detection in this study would be 6.5 cm, as markers were placed at 65 cm from the headset’s camera. However, even though both 8 × 8 and 12 × 12 cm markers are larger than the recommended size, the errors associated with them presented significant differences (Fig. 6 and Online Resource 13). In addition, 4 × 4 cm markers tended to shrink the virtual hexagons (i.e. to reduce their area), which might be explained by larger errors in the virtual hexagon’s inclination angle associated with this marker size (Fig. 6a), while 8 × 8 and 12 × 12 cm markers resulted in an expansion of the virtual hexagon (i.e. increased area). This might be partially because these marker sizes led to the virtual hexagons being rendered most often in front of the monitor’s surface (Online Resources 9 and 10), with larger distance-to-monitor errors than the 4 × 4 cm markers (Fig. 6b), and thus at a shorter distance to the headset’s camera than the distance predefined within the AR scene. Improvement in camera resolution may enhance marker detection and provide a more reliable registration regardless of marker position and size.

Having to use large markers at optimal positions relative to the headset’s camera to achieve ≤ 2° and ≤ 2 mm errors is not practical during surgery for two main reasons; (1) it would require that the surgeon wearing the headset has their head in a static predefined position; (2) marker sizes that are optimal for marker detection might not be optimal in terms of surgical needs, e.g. because they may occlude the surgeon’s view of the surgical site. To implement AR guiding systems in clinical practice, their performance must be optimised so that registration accuracy is not dependant on specific marker positions and sizes.

### Study limitations

Data are subject to bias derived from HoloLens' image processing as they were obtained from images captured by the headset’s camera. Some studies avoided this problem by measuring the accuracy with which participants trace the outlines of displayed virtual shapes on graph paper and on a small number of patients [36]. However, this approach includes additional sources of error, e.g. variations in the patient’s position. A single marker and position of the headset’s camera (i.e. Spots 1 or 2) were used for marker detection and thus all data points for registration computation laid in the same plane and were collected from a single point of view. This may have led to larger registration errors than if several markers and/or several positions of the headset’s camera had been used. In addition, having to move the headset between spots for marker detection and photograph-capture may have introduced a bias. Marker positions 1–8 may have caused larger errors compared to marker position 9 as the former were located at a distance of 108 mm from the centre of the digital graph chart while the latter was aligned with the centre (Fig. 2a). Hoff et al. [37] highlighted that markers should be placed as close as possible to the real-world feature with which the virtual model is to be registered to minimise registration errors. This recommendation was also supported by El-Hariri [38], who interpreted the large registration errors in their experiment as a result of small rotational errors in the detection of image markers placed far from the real-world feature. In addition, the x-axis inclination angle of the virtual hexagons may have introduced errors in the virtual hexagons’ position and dimensions. However, this variable was not considered in this study and thus errors derived from it were not detected. Furthermore, changes in the shape of the virtual hexagons were not analysed, although this information would have been useful to understand the deformations that they suffered. Finally, our experiments used a flat surface (i.e. the monitor’s surface), while virtual models are typically overlaid on a volumetric surface (i.e. the patient’s body surface) during surgery which may lead to larger registration errors.

## Conclusions

This study used a holographic headset combined with an image-pattern-recognition tool which provided ≤ 2° and ≤ 2 mm inclination angle and positional errors, respectively, in 70–75% of cases. In addition, it failed to provide submillimetre accuracy as required for high-precision surgical tasks such as the excision of small tumours [13]. Certain marker positions and sizes significantly increased the errors in the virtual hexagons’ position and dimensions. To make this technology reliable for clinical practice, sufficient accuracy with marker sizes that meet surgical needs regardless of marker position will be necessary.

## Supplementary Information

Below is the link to the electronic supplementary material.

Supplementary file1 (PDF 165 kb)

Supplementary file2 (PDF 105 kb)

Supplementary file3 (PDF 89 kb)

Supplementary file4 (PDF 56 kb)

Supplementary file5 (PDF 60 kb)

Supplementary file6 (PDF 157 kb)

Supplementary file7 (PDF 12 kb)

Supplementary file8 (PDF 506 kb)

Supplementary file9 (PDF 249 kb)

Supplementary file10 (PDF 228 kb)

Supplementary file11 (PDF 36 kb)

Supplementary file12 (PDF 139 kb)

Supplementary file13 (PDF 50 kb)

## Data Availability

Data supporting the findings of this study area available from the corresponding author (LP) upon reasonable request.
