# Supplementary material for: Effect of marker position and size on the registration accuracy of HoloLens in a non-clinical setting with implications for high-precision surgical tasks
Source: Int J Comput Assist Radiol Surg. 2021 Apr 15;16(6):955–66. doi: 10.1007/s11548-021-02354-9 (PMC8166698; doi:10.1007/s11548-021-02354-9)
Supplement: Supplementary file 2 — Supplementary file2 (PDF 105 kb) [file 11548_2021_2354_MOESM2_ESM.pdf]

Online Resource 10

Table S4 Number and percentage of cases in which the virtual hexagons appeared in front or behind the monitor's surface and tilted around their y-axis

		N	%
Position relative to monitor	Front	674	69.3
	Behind	298	30.7

[a]All marker positions (1-9) and 8x8 and 12x12 cm markers included (*n* = 972). Front and behind refer to the virtual hexagons being rendered in front of the monitor's surface and behind it, respectively

Title: Effect of marker position and size on the registration accuracy of HoloLens in a non-clinical setting with implications for high-precision surgical tasks

Journal: International Journal of Computer Assisted Radiology and Surgery

Authors: Laura Pérez-Pachón[1], Parivrudh Sharma[1], Helena Brech[1], Jenny Gregory[1], Terry Lowe[1,3], Matthieu Poyade[2], Flora Gröning[1]

[1] School of Medicine, Medical Sciences and Nutrition, University of Aberdeen, Aberdeen, United Kingdom

[2] School of Simulation and Visualisation, Glasgow School of Art, Glasgow, United Kingdom

[3] Head and Neck Oncology Unit, Aberdeen Royal Infirmary (NHS Grampian), Aberdeen, United Kingdom

Corresponding author: laura.perezpachon@gmail.com (LP)
