# Supplementary material for: Effect of marker position and size on the registration accuracy of HoloLens in a non-clinical setting with implications for high-precision surgical tasks
Source: Int J Comput Assist Radiol Surg. 2021 Apr 15;16(6):955–66. doi: 10.1007/s11548-021-02354-9 (PMC8166698; doi:10.1007/s11548-021-02354-9)
Supplement: Supplementary file 3 — Supplementary file3 (PDF 89 kb) [file 11548_2021_2354_MOESM3_ESM.pdf]

Online Resource 11

Table S5 Vertex position, inclination angle, distance-to-monitor, centroid and area errors for the 12x12 and 8x8 cm marker sizes (n = 5832) with marker positions 1-9

	Marker position	N	Min	Max	Mean	SD
Inclination angle (°)	1	108	2.2	7.1	4.8	1.2
	2	108	4.6	9.9	6.8	1.5
	3	108	1.7	5.7	3.7	0.9
	4	108	0	4.3	1.3	1.1
	5	108	1.7	9.2	5.2	1.7
	6	108	4.9	13.6	8.5	1.6
	7	108	2.9	9.9	5.5	1.6
	8	108	0	2.1	0.7	0.5
	9	108	0	3.3	1.3	0.8
Distance-to-monitor (mm)	1	108	0.7	19.9	11.0	4.9
	2	108	0	6.1	2.4	1.8
	3	108	0.5	15.0	6.5	3.8
	4	108	2.9	32.9	12.7	9.7
	5	108	0	31.0	11.1	8.9
	6	108	0.2	13.2	7.4	3.3
	7	108	3.7	23.4	13.7	5.4
	8	108	12.2	30.6	21.7	4.4
	9	108	6.0	16.6	11.0	2.7
Vertex position (mm)	1	648	0.1	3.9	1.5	0.7
	2	648	0	3.5	1.1	0.5
	3	648	0.4	8.1	1.8	0.8
	4	648	0.1	14.8	2.9	3.1
	5	648	0	12.5	3.1	3.3
	6	648	0.8	7.8	3.3	1.5
	7	648	0.1	12.8	2.9	2.4
	8	648	0.1	8.9	1.7	1.4
	9	648	0	2.0	0.8	0.4

Centroid position (mm)		1	108	0.5	2.0	1.1	0.4
		2	108	0.2	1.5	0.8	0.3
		3	108	0.5	3.2	1.3	0.5
		4	108	0.9	9.0	2.8	2.7
		5	108	0.2	7.4	2.7	2.6
		6	108	1.8	3.9	2.8	0.5
		7	108	0.3	6.6	2.3	1.8
		8	108	0.3	4.9	1.3	1.1
		9	108	0	0.8	0.4	0.2
Area (%)	Absolute	1	108	0	3.8	1.1	0.8
		2	108	0.1	3.4	1.5	0.9
		3	108	0	5.5	1.7	1.1
		4	108	0	15.5	4.5	4.8
		5	108	0	12.5	4.3	4.6
		6	108	1.2	8.0	4.8	1.2
		7	108	0	14.1	3.9	3.7
		8	108	0.1	11.3	4.0	2.4
		9	108	0	4.7	1.7	0.9
	Relative	1	108	-2.2	3.8	0.7	1.1
		2	108	-3.4	2.1	-1.3	1.1
		3	108	-3.8	5.5	1.6	1.3
		4	108	-15.5	4.9	-1.7	6.4
		5	108	-12.5	4.3	-2.9	5.6
		6	108	-8.0	-1.2	-4.8	1.2
		7	108	-14.1	2.6	-3.0	4.5
		8	108	-11.3	5.8	2.2	4.1
		9	108	-0.6	4.7	1.7	0.9

Title: Effect of marker position and size on the registration accuracy of HoloLens in a non-clinical setting with implications for high-precision surgical tasks

Journal: International Journal of Computer Assisted Radiology and Surgery

Authors: Laura Pérez-Pachón[1], Parivrudh Sharma[1], Helena Brech[1], Jenny Gregory[1], Terry Lowe[1,3], Matthieu Poyade[2], Flora Gröning[1]

[1] School of Medicine, Medical Sciences and Nutrition, University of Aberdeen, Aberdeen, United Kingdom

[2] School of Simulation and Visualisation, Glasgow School of Art, Glasgow, United Kingdom

[3] Head and Neck Oncology Unit, Aberdeen Royal Infirmary (NHS Grampian), Aberdeen, United Kingdom

Corresponding author: laura.perezpachon@gmail.com (LP)
