# Supplementary material for: Effect of marker position and size on the registration accuracy of HoloLens in a non-clinical setting with implications for high-precision surgical tasks
Source: Int J Comput Assist Radiol Surg. 2021 Apr 15;16(6):955–66. doi: 10.1007/s11548-021-02354-9 (PMC8166698; doi:10.1007/s11548-021-02354-9)
Supplement: Supplementary file 4 — Supplementary file4 (PDF 56 kb) [file 11548_2021_2354_MOESM4_ESM.pdf]

Online Resource 12

Table S6 Mean errors for all dependant variables including 8x8 and 12x12 cm markers

		Marker size	N	Min	Max	Mean	SD
Inclination angle (°)		8	486	0	13.6	4.6	3.1
		12	486	0	8.7	3.7	2.4
Distance-to-monitor (mm)		8	486	0	32.9	12.0	8.3
		12	486	0	27.7	9.6	6.5
Vertex position (mm)		8	2916	0	14.8	2.6	2.6
		12	2916	0	9.0	1.6	1.3
Centroid position (mm)		8	486	0	9.0	2.2	2.1
		12	486	0	4.7	1.2	0.9
Area (%)	Absolute	8	486	0	15.5	3.7	3.8
		12	486	0	9.8	2.4	1.9
	Relative	8	486	-15.5	5.8	-1.9	5
		12	486	-9.8	5.5	0.2	3

Title: Effect of marker position and size on the registration accuracy of HoloLens in a non-clinical setting with implications for high-precision surgical tasks

Journal: International Journal of Computer Assisted Radiology and Surgery

Authors: Laura Pérez-Pachón[1], Parivrudh Sharma[1], Helena Brech[1], Jenny Gregory[1], Terry Lowe[1,3], Matthieu Poyade[2], Flora Gröning[1]

[1] School of Medicine, Medical Sciences and Nutrition, University of Aberdeen, Aberdeen, United Kingdom

[2] School of Simulation and Visualisation, Glasgow School of Art, Glasgow, United Kingdom

[3] Head and Neck Oncology Unit, Aberdeen Royal Infirmary (NHS Grampian), Aberdeen, United Kingdom

Corresponding author: laura.perezpachon@gmail.com (LP)
