# Supplementary material for: Effect of marker position and size on the registration accuracy of HoloLens in a non-clinical setting with implications for high-precision surgical tasks
Source: Int J Comput Assist Radiol Surg. 2021 Apr 15;16(6):955–66. doi: 10.1007/s11548-021-02354-9 (PMC8166698; doi:10.1007/s11548-021-02354-9)
Supplement: Supplementary file 5 — Supplementary file5 (PDF 60 kb) [file 11548_2021_2354_MOESM5_ESM.pdf]

Online Resource 13

Table S7 Mean errors for all dependant variables including 4x4, 8x8 and 12x12 cm markers and marker position 9

		Marker size	N	Min	Max	Mean	SD
Inclination angle (°)		4	54	2.3	6.3	3.7	0.9
		8	54	0.3	3.3	1.3	0.9
		12	54	0	2.6	1.2	0.7
Distance-to-monitor (mm)		4	54	1.4	6.7	4.0	1.4
		8	54	6.0	16.6	10.2	3.2
		12	54	9.6	15.8	11.8	1.9
Vertex position (mm)		4	324	0.0	5.7	1.3	0.8
		8	324	0	1.7	0.7	0.4
		12	324	0.1	2.0	0.9	0.4
Centroid position (mm)		4	54	0.1	2.6	1.2	0.7
		8	54	0	0.7	0.3	0.2
		12	54	0	0.8	0.5	0.2
Area (%)	Absolute	4	54	0	3.6	1.2	0.9
		8	54	0	3.3	1.4	0.9
		12	54	0.6	4.7	2.0	0.9
	Relative	4	54	-3.6	1.0	-1.1	1.1
		8	54	-0.6	3.3	1.3	0.9
		12	54	0.6	4.7	2.0	0.9

Title: Effect of marker position and size on the registration accuracy of HoloLens in a non-clinical setting with implications for high-precision surgical tasks

Journal: International Journal of Computer Assisted Radiology and Surgery

Authors: Laura Pérez-Pachón[1], Parivrudh Sharma[1], Helena Brech[1], Jenny Gregory[1], Terry Lowe[1,3], Matthieu Poyade[2], Flora Gröning[1]

[1] School of Medicine, Medical Sciences and Nutrition, University of Aberdeen, Aberdeen, United Kingdom

[2] School of Simulation and Visualisation, Glasgow School of Art, Glasgow, United Kingdom

[3] Head and Neck Oncology Unit, Aberdeen Royal Infirmary (NHS Grampian), Aberdeen, United Kingdom

Corresponding author: laura.perezpachon@gmail.com (LP)
