# Supplementary material for: Effect of marker position and size on the registration accuracy of HoloLens in a non-clinical setting with implications for high-precision surgical tasks
Source: Int J Comput Assist Radiol Surg. 2021 Apr 15;16(6):955–66. doi: 10.1007/s11548-021-02354-9 (PMC8166698; doi:10.1007/s11548-021-02354-9)
Supplement: Supplementary file 7 — Supplementary file7 (PDF 12 kb) [file 11548_2021_2354_MOESM7_ESM.pdf]

Online Resource 3

Table S1 Intra-class correlation coefficient of researchers 1-3 in the measurements of the vertex alignment, inclination angle and distance-to-monitor extracted from the photos

	Researcher	ICC
Inclination angle	1	0.979
	2	0.979
	3	0.953
Distance-to-monitor	1	0.994
	2	0.989
	3	0.975
Vertex position	1	0.998
	2	0.995
	3	0.956

Title: Effect of marker position and size on the registration accuracy of HoloLens in a non-clinical setting with implications for high-precision surgical tasks

Journal: International Journal of Computer Assisted Radiology and Surgery

Authors: Laura Pérez-Pachón[1], Parivrudh Sharma[1], Helena Brech[1], Jenny Gregory[1], Terry Lowe[1,3], Matthieu Poyade[2], Flora Gröning[1]

[1] School of Medicine, Medical Sciences and Nutrition, University of Aberdeen, Aberdeen, United Kingdom

[2] School of Simulation and Visualisation, Glasgow School of Art, Glasgow, United Kingdom

[3] Head and Neck Oncology Unit, Aberdeen Royal Infirmary (NHS Grampian), Aberdeen, United Kingdom

Corresponding author: laura.perezpachon@gmail.com (LP)
