# Supplementary material for: Effect of marker position and size on the registration accuracy of HoloLens in a non-clinical setting with implications for high-precision surgical tasks
Source: Int J Comput Assist Radiol Surg. 2021 Apr 15;16(6):955–66. doi: 10.1007/s11548-021-02354-9 (PMC8166698; doi:10.1007/s11548-021-02354-9)
Supplement: Supplementary file 11 — Supplementary file11 (PDF 36 kb) [file 11548_2021_2354_MOESM11_ESM.pdf]

Online Resource 7

Table S2 Strong outliers for each dependant variable including their percentage and lower and upper ranges

		Lower range		Upper range		Percentage	N
		Min	Max	Min	Max		
Inclination angle (°)		-	-	18.8	-	0%	0
Distance-to-monitor (mm)		-	-	22.1	9	10.1%	98
Vertex position (mm)		-	-	4.4	14.8	10.2%	592
Centroid position (mm)		-	-	3.5	9	12.6%	122
Area (%)	Absolute	-	-	12.5	15.5	1.3%	13
	Relative	-15.5	-14.9	14.5	-15.1	0.5%	5

Title: Effect of marker position and size on the registration accuracy of HoloLens in a non-clinical setting with implications for high-precision surgical tasks

Journal: International Journal of Computer Assisted Radiology and Surgery

Authors: Laura Pérez-Pachón[1], Parivrudh Sharma[1], Helena Brech[1], Jenny Gregory[1], Terry Lowe[1,3], Matthieu Poyade[2], Flora Gröning[1]

[1] School of Medicine, Medical Sciences and Nutrition, University of Aberdeen, Aberdeen, United Kingdom

[2] School of Simulation and Visualisation, Glasgow School of Art, Glasgow, United Kingdom

[3] Head and Neck Oncology Unit, Aberdeen Royal Infirmary (NHS Grampian), Aberdeen, United Kingdom

Corresponding author: laura.perezpachon@gmail.com (LP)
